# Segmental range-of-motion by vertebral level in fused and unfused patients with adolescent idiopathic scoliosis: a systematic review of the literature

**DOI:** 10.1007/s43390-024-00978-w

**Published:** 2024-09-29

**Authors:** Omkar S. Anaspure, Anthony N. Baumann, Marc T. Crawford, Pierce Davis, Laura C. M. Ndjonko, Jason B. Anari, Keith D. Baldwin

**Affiliations:** 1 Perelman School of Medicine, University of Pennsylvania, Philadelphia, PA USA; 2 College of Medicine, Northeast Ohio Medical University, Rootstown, OH USA; 3 Department of Physical Therapy, University of North Texas Health Science Center, Fort Worth, TX USA; 4 Department of Biological Sciences, Northwestern University, Chicago, IL USA; 5 Department of Orthopedic Surgery, Children’s Hospital of Philadelphia, 3401 Civic Center Blvd, Philadelphia, PA 19140 USA

**Keywords:** Adolescent idiopathic scoliosis, Segmental range-of-motion, Vertebral motion, Pediatric spine

## Abstract

**Purpose:**

This study aims to understand global and segmental spinal ROM in surgical and nonsurgical AIS patients.

**Methods:**

This systematic review examined segmental vertebral ROM in AIS patients using PubMed, SPORTDiscus, MEDLINE, and Web of Science until October 8th, 2023. Inclusion criteria were articles on segmental motion in AIS patients, both operative and non-operative, under 18 years old.

**Results:**

Seventeen articles met eligibility criteria from 2511 initially retrieved. All patients (*n* = 996) had AIS (549 non-operative; 447 were operative), with a frequency-weighted mean age of 15.1 ± 1.6 years and a baseline Cobb angle of 51.4 ± 13.3 degrees. Studies showed heterogenous segmental flexibility in the unfused spine, with the apical curve and upper thoracic segments being more rigid and lower segments more flexible at -5 disk segments from the apex. Most studies showed a predictable loss of motion in fused spinal regions postoperatively and a variable loss of global motion depending on the LIV and number of fused segments. A 7° global loss of total trunk flexion per level was observed with increasingly caudal LIV, starting at L1. Anterior vertebral body tethering (AVBT) preserved motion post-surgery but reduced coronal plane motion. AVBT saw less motion loss compared to posterior spinal fusion (PSF) but had higher revision and complication rates.

**Conclusion:**

Preservation of spinal segments correlated with improved motion postoperatively. Increasing caudal LIV in PSF showed sagittal flexion loss. AVBT preserved more sagittal ROM than PSF but increased coronal motion loss, complications, and revision rates, with the largest benefit at LIV L4. Data on segmental motion are limited and further research on postoperative segmental ROM is required.

## Introduction

Adolescent idiopathic scoliosis (AIS) is the most common pediatric spinal deformity, characterized by a predominantly coronal curvature [1]. AIS affects approximately 0.47–5.2% of adolescents and varies in severity, with potential progression during growth [2]. Once scoliotic curves reach a coronal curve of 50-degrees, the natural history is a relentless slow progression in adulthood of 0.5° to 1° per year [3]. Surgical intervention is recommended to prevent further progression and avoid potential future disability and deformity [4]. The gold standard treatment is posterior spinal fusion, with anterior vertebral body tethering (AVBT) as a newer motion-sparing option [5]. Goals of surgery are to arrest the progression of the curve and secondarily to provide correction of the 3- dimensional deformity [6, 7]. Instrumented fusion provides superior correction but eliminates motion in fused segments [8]. More distal lumbar fusions are seen with increased pain and revision surgery rates, along with lowering health-related quality of life [9]. Understanding fusion consequences is crucial when considering AVBT, which has a higher published complication and revision rate than the gold standard posterior spinal fusion.

Global ROM refers to overall spinal flexibility, while segmental ROM focuses on individual spinal segments, often overlooked in the literature [10, 11]. Understanding how the ROM of the spine in AIS behaves, with or without surgical intervention, is an area that requires deeper investigation [12–15]. Despite a wealth of research on AIS and possible surgical interventions, comprehensive studies on segmental ROM in AIS patients are lacking on a large scale [5, 7, 8, 16–20]. Current literature mainly addresses global ROM, often neglecting how individual segments contribute to overall motion [10, 21, 22]. Though previous reviews have investigated global ROM, none have synthesized information on segmental ROM in scoliosis [10, 23]. This paper aims to evaluate and synthesize existing literature on segmental ROM in unfused segments, with and without instrumentation, in both surgical and nonsurgical patients. Understanding global and segmental ROM in AIS surgery aids treatment planning, surgical decision-making, patient counseling, and postoperative management [24–28].

## Methods

### Study creation and initial search

This study is a qualitative systematic review of the literature examining segmental vertebral ROM in patients with AIS using PubMed, SPORTDiscus, MEDLINE, and Web of Science from database inception until October 8th, 2023. Search terms used in each database were: “scoliosis” AND (pediatric OR adolescent OR children) AND (“range-of-motion” OR “range of motion” OR mobility OR motion) AND (segment OR segmental OR spinal OR spine OR vertebral). This study was performed under the guidelines of the most recent Preferred Reporting Items for Systematic Reviews and Meta-Analyses (PRISMA) for proper data reporting.

### Inclusion and exclusion criteria

Inclusion criteria were articles that examined segmental (single or several vertebral levels) motion with or without global ROM measurements, patients with AIS, operative or non-operative patients, pediatric patients (< 18 years old), observational studies, randomized controlled trials, and full-text English articles. Exclusion criteria were studies that only measured total spinal motion (i.e. forward flexion of the entire spine), patients without AIS, adult patients (> 18 years old), systematic reviews, books, case reports, and no full text.

### Article screening process

After the search algorithm was used in each of the four databases for the initial search, all of the articles were downloaded into Rayyan, a public website used for systematic reviews. Duplicate articles were then manually removed and the remaining articles were sorted by title and abstract via the inclusion and exclusion criteria. Next, the remaining articles underwent a full-text search for final study inclusion. The article screening process was completed by two authors in this systematic review.

### Data extraction

Data extraction was completed by a single author. Data extracted included first author, year of publication, diagnosis (AIS), type of patient (operative or non-operative), type of surgery (if operative patient), number of patients, average age, average follow-up, baseline Cobb angle (degrees), Risser stage, and relevant qualitative data with associated *p*-values for narrative reporting.

### Article quality grading

All included articles were graded for quality via the Methodological Index for Non-Randomized Studies (MINORS) scale, a commonly used grading tool for observational studies in the literature. Based on the MINORS scale, articles were classified into comparative studies (out of 0–24 points) or non-comparative studies (out of 0–16 points). For this study, the total MINORS score was used to classify articles as high quality (24 points for comparative studies and 16 points for non-comparative studies), moderate quality (15–23 points for comparative studies and 10–15 points for non-comparative studies), or low quality (< 15 points for comparative studies and < 10 for non-comparative studies) based on precedent in the existing literature.

### Statistical analysis

This study utilized the Statistical Package for the Social Sciences (SPSS) version 29.0 (Armonk, NY: IBM Corp) for statistical analysis. Frequency-weighted means and other descriptive statistics were used to describe the data where no statistical significance could be calculated. No formal meta-analysis was undertaken for this study due to study heterogeneity. Therefore, a narrative approach for qualitative systematic review was considered the best way to represent the data available for this topic.

## Results

### Initial study results

**Fig. 1:**
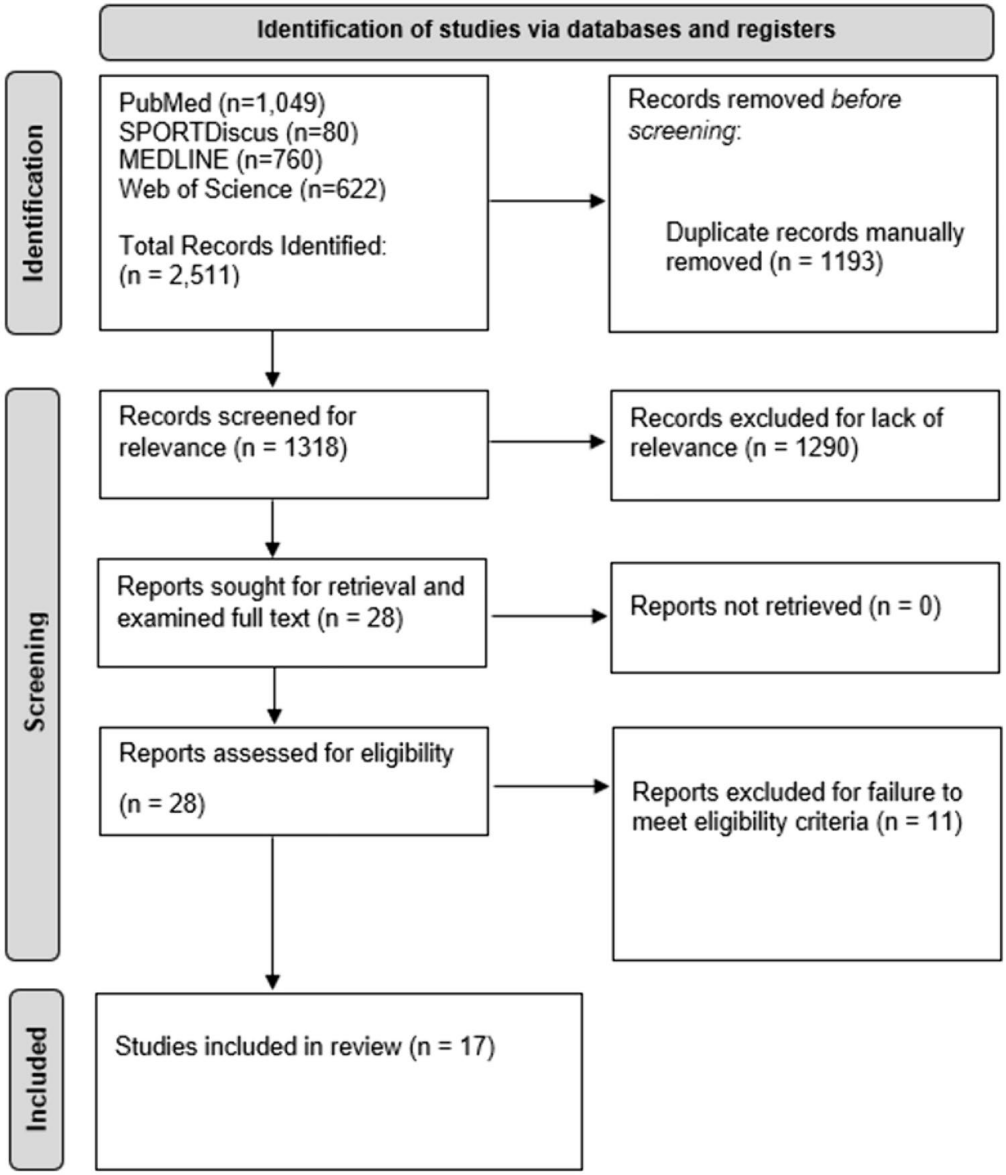
The preferred reporting items for systematic reviews and meta-analyses (PRISMA) diagram for this study outlining initial search, article screening, and final article inclusion

A total of 17 articles met eligibility criteria from 2511 articles initially retrieved from the four databases used in this systematic review (Fig. 1). All included articles were observational studies and were graded via the MINORS scale (Table 1). Notably, no randomized controlled trials were found for this systematic review. Four articles were comparative studies with two classified as low quality and two classified as moderate quality based on the MINORS scale. Of the remaining 13 non-comparative articles, four articles were classified as low quality, and nine articles were classified as moderate quality. No high-quality articles were found for this systematic review. In total, this study included six low-quality articles and 11 moderate-quality articles.

**Table 1:** The Methodological Index for Non-Randomized Studies (MINORS) grading for the individual articles included in this systematic review. Comparative studies are out of 0–24 points and non-comparative studies are out of 0–16 points

Author (year)	Study type	Total minors score	Clearly stated aim	Inclusion of consecutive patients	Prospective collection of data	End points appropriate to study aim	Unbiased assessment of study end point	Follow-up period appropriate to study aim	Less than 5% lost to follow up	Prospective calculation of the study size	Adequate control group	Contemporary groups	Baseline equivalence of groups	Adequate statistical analysis
Hosseini (2016)	Non-comparative	14	2	2	2	2	0	2	2	2	–	–	–	–
Chan (2016)	Non-comparative	11	2	2	2	2	0	0	1	2	–	–	–	–
Yao (2017)	Non-comparative	14	2	2	2	2	0	2	2	2	–	–	–	–
Fan (2016)	Non-comparative	10	2	2	0	2	0	2	2	0	–	–	–	–
Hasler (2010)	Non-comparative	7	2	2	0	2	0	0	1	0	–	–	–	–
Hirsch (2016)	Non-comparative	11	2	2	2	2	0	0	1	2	–	–	–	–
Kao (2014)	Non-comparative	7	2	2	0	2	0	0	1	0	–	–	–	–
Hoyek (2023)	Non-comparative	7	2	2	0	2	0	0	1	0	–	–	–	–
Sung (2020)	Comparative	21	2	2	2	2	2	0	1	2	2	2	2	2
Jamison (2022)	Comparative	21	2	2	2	2	0	2	1	2	2	2	2	2
Buyuk (2021)	Non-comparative	14	2	2	2	2	0	2	2	2	–	–	–	–
Marks (2012)	Non-comparative	14	2	2	2	2	0	2	2	2	–	–	–	–
Engsberg (2002)	Non-comparative	14	2	2	2	2	0	2	2	2	–	–	–	–
Xiong (1993)	Non-comparative	11	2	2	2	2	0	0	1	2	–	–	–	–
Kawasaki (2020)	Non-comparative	8	2	2	0	2	0	1	1	0	–	–	–	–
Pahys (2022)	Comparative	13	2	2	0	2	0	2	1	0	0	0	2	2
Marks (2015)	Comparative	14	2	2	0	2	0	2	1	2	0	0	2	1

### Patient demographics

All included patients (*n* = 996) had a diagnosis of AIS with a frequency-weighted mean age of 15.1 ± 1.6 years (*n* = 996) and a baseline Cobb angle of 51.4 ± 13.3 degrees (*n* = 792). Of the total 996 patients, 549 patients were non-operative patients (no surgery was performed) and 447 patients were operative patients (surgery was performed). Non-operative patients (*n* = 549) had a frequency-weighted mean age of 14.8 ± 1.0 years (*n* = 549) and a baseline Cobb angle of 47.6 ± 17.5 degrees (*n* = 417). Operative patients (*n* = 447) had a frequency-weighted mean age of 15.4 ± 2.0 years (*n* = 447) and a baseline Cobb angle of 55.7 ± 1.6 degrees (*n* = 375). Demographic information and study characteristics from each of the individual included articles can be seen in Table 2.

**Table 2:** Patient demographics from the individual studies included in this systematic review. Data recorded includes first author, year of publication, operative or non-operative, patient diagnosis, number of patients, average patient age, baseline Cobb angle (preoperative if surgical patient), Lenke curve classification, follow-up information, and Risser stage information

First author (publication year)	Operative status	Type of surgery	Patient diagnosis	Patients (n)	Mean patient age	Baseline Cobb angle (degrees)	Lenke curve	Mean postoperative follow-up	Risser 0	Risser 1	Risser 2	Risser 3	Risser 4	Risser 5
Jamison (2022)	Non-operative	–	Adolescent idiopathic scoliosis	53	13.34 ± 1.25	28.24 ± 10.24	–	–	7	0	4	6	8	2
Buyuk (2021)	Operative	Thoracic anterior vertebral body tethering	Adolescent idiopathic scoliosis	32	13	51	–	2 years	19	7	6	–	–	–
Hoyek (2023)	Non-operative	–	Adolescent idiopathic scoliosis	18	16	38	–	–	–	–	–	–	–	–
Yao (2017)	Operative	Alternate-level pedicle screw fixation	Adolescent idiopathic scoliosis	80	15 ± 3	57	47 Type 1, 33 type 4	2 years	–	–	–	–	–	–
Sung (2020)	Non-operative	–	Adolescent idiopathic scoliosis	14	14.79 ± 1.05	22.01 ± 4.99	–	–	–	–	–	–	–	–
Chan (2016)	Non-operative	–	Adolescent idiopathic scoliosis	100	15.3	67.5 ± 18.1	33 Lenke 1-ve, 37 Lenke 1 + ve, 30 Lenke 2	–	–	–	–	–	–	–
Hasler (2010)	Non-operative	–	Adolescent idiopathic scoliosis	76	14.9	59.7 ± 12.9	–	–	–	–	–	–	–	–
Kawasaki (2020)	Non-operative	–	Adolescent idiopathic scoliosis	21	15.5	58.24 ± 2.69	–	–	–	–	–	–	–	–
Hirsch (2016)	Non-operative	–	Adolescent idiopathic scoliosis	50	15.6 ± 1.9	53.4 ± 12.3	23 Lenke 1, 10 Lenke 2, 4 Lenke 3, 4 Lenke 4, 6 Lenke 5, 3 Lenke 6	–	–	–	–	–	–	–
Xiong (1993)	Non-operative	–	Adolescent idiopathic scoliosis	132	13.7	–	–	–	–	–	–	–	–	–
Marks (2015)	Operative	Spinal Fusion	Adolescent idiopathic scoliosis	259	14 ± 2	–	134 Lenke 1, 51 Lenke 2, 9 Lenke 3, 10 Lenke 4, 40 Lenke 5	–	–	–	–	–	–	–
Pahys (2022)	Operative	Anterior vertebral body tethering, posterior spinal fusion (pre and post op)	Adolescent idiopathic scoliosis	112	13.2	56.27	78 Lenke 1, 19 Lenke 2, 6 Lenke 3, 1 Lenke 4, 4 Lenke 5, 4 Lenke 6	–	–	–	–	–	–	–
Marks (2012)	Operative	Spinal Fusion	Adolescent idiopathic scoliosis	100	15 ± 2	55 ± 10	64 Lenke 1 curves, 12 Lenke 2 curves, 6 Lenke 3 curves, 1 Lenke 4 curve, 15 Lenke 5 curves, and 2 Lenke 6 curve type	3.3 ± 1.3 years	–	–	–	–	–	–
Kao (2014)	Non-operative	–	Adolescent idiopathic scoliosis	85	16.1 ± 2.84	25.56 ± 11.61	–	–	6	2	0	7	17	53
Fan (2016)	Operative	Spinal Fusion	Adolescent idiopathic scoliosis	172	17.8	–	–	2.475 years	–	–	–	–	–	–
Engsberg (2002)	Operative	Spinal Fusion	Adolescent idiopathic scoliosis	30	14	57	–	1 and 2 year intervals	–	–	–	–	–	–
Hosseini (2016)	Operative	Apical short-segment correction technique	Adolescent idiopathic scoliosis	21	14.2 ± 1.5	56.1	20 Lenke 1a, 1 Lenke 1B	2 weeks, 3, 6, and 1 year intervals	2	3	1	2	8	5

### Thoracic spine segmental range-of-motion and characteristics in unfused scoliotic spines

Six studies included patients who had unfused scoliotic spines. Hoyek et al. (2023) sought to investigate trunk movements in AIS patients via radiologic examination [29]. AIS patients showed decreased ROM of the thoracic segments (*p* < 0.05) with all trunk movements such as flexion, extension, lateral bending, and rotation of the trunk compared to normal age and sex-matched controls. However, there were no significant limitations in terms of trunk extension and lateral bending motions. The findings of Sung et al. (2020) built on this by comparing spinal ROM between patients with right convex thoracic AIS and control subjects. A moderate positive correlation was noted between the severity of the spinal curve (Cobb angle) and ROM in the mid-thorax region (T7) in those with AIS (*r* = 0.62, *p* = 0.04). [30]. Chan et al. (2016) investigated proximal thoracic (PT) flexibility and compensatory ability above the potential upper instrumented vertebra (UIV) in 100 patients with Lenke 1 and 2 curves using cervical supine side-bending radiographs. [31]. They observed that Lenke 2 curves exhibited considerable stiffness, with over 80% of cases showing limited motion and failing to compensate between T3 and T6 (i.e. the proximal segment was stiff). A significant difference in segment motion was observed between “stiff” and “flexible” Lenke 1 curves from T3 to T6. Curves were designated as flexible if the left side bending angle is “0 degree” or “negative value”, meaning that the flexibility of the proximal thoracic curve will allow the C7 centroid to realign back to the center sacral vertical line. Curves were designated as stiff if the left side bending angle is a “positive value”, deeming that the proximal thoracic segment is deemed to be unable to compensate. A greater number of stiff Lenke 1 curves failed to show compensatory motion compared to flexible Lenke 1 curves (*p* < 0.05). Chan et al. (2016) ultimately suggested that flexible Lenke 1 curves retain better segmental motion and flexibility in the PT regions, while Lenke 2 curves and stiff Lenke 1 curves are less capable of compensatory movement and exhibit greater stiffness. Hasler et al. (2010) calculated a flexibility index by comparing segmental standing and fulcrum bending disc angles in 76 AIS patients [32]. Hasler et al. (2010) found the average flexibility of the curves measured to be about 49%. Table 3 includes the flexibility indices of other studies reviewed. Flexibility varied significantly, with decreasing flexibility close to the apex and increasing flexibility noted distal to the apex. Specifically, when comparing the flexibility at distal and proximal segments, the segments immediately adjacent to the apex of the curve were significantly less flexible (46% to 49% flexible), whereas segments further from the apex showed much greater flexibility (105% to 300%) (*p* < 0.001). Hasler et al. also found that both the discs and bones of the spine contributed to the curvature, with discs contributing 60% and bones 40% (*p* < 0.0001). The data suggest that the most severe motion loss is limited to a few specific segments near the curve’s apex. Kawasaki et al. (2020) measured the segmental flexibility by calculating the Flexiblity Index (FI) for each disk level in unfused AIS spines. They defined the curve apex vertebra as level “0”, with the cranial disk levels increasingly defined as “ + 1” to “ + 5”, and the descending caudal levels as “ − 1” to “ − 5”. The group found the greatest segmental flexibility was seen at the level − 5 disk (median FI, 50%) [17]. Kawasaki et al. (2020) also found segments cranial to the apex (+ 5 segments) to have an FI ranging from 50 to 75, whereas segments caudal to the curve apex (-5 segments) have FI values of 66.7 to 150 (*p* < 0.05) [17]. This suggests that in AIS patients, the upper and middle segments of the thoracic spine are stiffer or less flexible compared to the lower segments. Additionally, the least flexibility was seen in the periapical segments (+ 1 and − 1; FI, 50%–66.7%) [17]. This may imply that apical and thoracic curvature segments are relatively rigid in affected untreated spines, which is specific to the AIS population studied by Kawasaki et al. (2020), and it is crucial to note this observation cannot be generalized to non-scoliotic spines in the absence of a control group. Xiong et al. (1993) supplemented this finding by showing that scoliotic patients had the most pronounced vertebral rotation angle (VRA) in apical segments [20]. It is helpful to note that there was also a significant correlation between VRA and increasing Cobb angle (*p* < 0.05).

**Table 3:** Flexibility index (FI) scores measured by radiographic imaging and forward flexion scores in unfused AIS spines from eight studies

Author	Patients	Flexibility index (%)	Forward flexion (degrees)
Chan (2016)	100	51.77 ± 13.07	–
Hasler (2010)	76	48.6 ± 14.3	–
Hosseini (2016)	21	51.1 ± 11.7	–
Hoyek (2023)	18	–	21
Jamison (2022)	53	–	17.92 ± 9.69
Kao (2014)	85	–	25.56 ± 12.33
Kawasaki (2020)	21	53.8 ± 3.3	-
Fan	172	–	41.4 ± 20.7
Yao (2017)	80	63 ± 16	–
Total	454	298	156

### Lumbar range-of-motion in unfused scoliotic spines

Three studies focused on lumbar flexibility in AIS patients with unfused spines. Sung et al. (2020) compared patients with and without AIS to examine motion. ROM measurements were taken at different points along the spine. Coupling Angles (CA) were also measured. This refers to the angle relationships between movements of different spine segments during rotation. Sung et al. (2020) found that in AIS patients, these CAs showed that different spine segments moved more independently from one another compared to controls, especially in the lumbar region of AIS patients (*p* = 0.02). Body mass index (BMI) demonstrated a moderate relationship between both patients and controls. There was less motion seen in the lumbar spine for patients with a high BMI (*r* =  − 0.67, *p* = 0.02) in the AIS group. Controls with a high BMI had more motion in the lower thorax (*r* = 0.59, *p* = 0.01) [30]. Similarly, Kao et al. (2014) focused on global spine ROM by investigating forward flexibility in the lumbar region in AIS patients in relation to increasing coronal plane deformity and its impact on mobility [33]. The magnitude of lumbar flexibility, as measured by a sit-and-reach (SR) test, was seen to have a negative correlation with increasing lumbar curvature from L1-L5 as defined by the Cobb angle. The correlation between the SR test and L1/L5 curves was negative (r_p_ =  − 0.595), implying that patients with larger lumbar curves in non-operative scoliosis patients tend to have stiffer lumbar spines. Jamison et al. (2022) sought to measure specifically lumbopelvic motion in AIS patients using an external ViMove DorsaVi sensor [34]. They found that when sitting, female AIS patients had an anterior ROM of − 5.17° versus 11.22° for controls (*n* = 18) (*p* = 0.0010), and a posterior ROM of − 27.61° versus − 18.83° for controls (*p* = 0.0232). This brings yet another dimension to the differences between patients with AIS and controls, as the findings demonstrate significant differences in lumbopelvic posture and ROM between the two groups.

### Range-of-motion at unfused levels in fused spines

Seven studies assessed spinal range of motion (ROM) following spinal fusion surgery. Notably, these studies present a complex picture of the effects of fusion on the motion of distal unfused segments. Marks et al. (2012) examined intervertebral segmental and cumulative motion in the distal unfused segments of 100 patients with adolescent idiopathic scoliosis (AIS) after thoracolumbar spinal fusion [18]. They found that distally instrumented vertebrae exhibited significantly greater L2–L3, L3–L4, and L4–L5 segmental motion during lateral bending, with motion increasing as the fusion extended more distally (*p* = 0.002, 0.009, and 0.001, respectively). This suggests that more distal fusions may allow greater compensatory motion in the segments below the fusion. The summed motion from L3 to S1 also increased with a more distal fusion (*p* = 0.001). Continuing this research, Marks et al. (2015) evaluated segmental coronal motion in the unfused distal segments (L4-S1) of 259 patients who underwent instrumented fusion across a 10-year follow-up [19]. The motion remained largely unchanged over time, but there was a notable 50% increase in summed lateral bending motion from L4 to S1 in patients with a lower instrumented vertebra (LIV) at L2 or lower compared to those with an LIV at L1 or higher. This supports the notion of increased compensatory mobility in segments distal to more extensive fusions. Fan et al. (2016) measured segmental lumbar spine ROM by LIV in 172 patients. The study demonstrated that the spinal range of motion (ROM) in patients with idiopathic scoliosis treated by spinal fusion varies significantly with the lower instrumented vertebra (LIV). In contrast to the increasing lateral bending found by Marks et al. (2012), Fan et al. found that spinal ROM, especially forward flexion, decreased significantly as the LIV extended more distally. Statistically significant (*p* < 0.001) differences in ROM measures were observed across different fusion groups, with the highest ROM observed in patients with the least distal LIVs, indicating that preserving motion segments above L2 can maintain greater lumbar mobility [25]. Pahys et al. (2022) assessed segmental trunk motions in 23 AIS patients postoperatively after PSF and 48 patients who underwent AVBT [35]. Contrary to Marks et al. (2012, 2015), this study found PSF patients to have a significant loss of motion in all 4 directions at 2 years postoperatively (flexion loss was 11° for ≤ L1 to 30° for L4; *p* < 0.001) and a loss of global trunk flexion 2 years after PSF compared to AVBT. There was a significant and consistent loss of thoracic extension 2 years after PSF in all LIV groups (loss of 11° for ≤ L1 to 30° for L4; *p* < 0.001 for both). This cumulatively can be summed as a 7° global loss of flexion per additional lumbar level of LIV. The 48 AVBT patients only demonstrated a loss of flexion and side-bending at 2 years postoperatively such as a flexion loss of 11° for L1 to 17° for L4 (*p* < 0.001) [35]. No differences were seen in thoracic VBT vs thoracic PSF. This suggests that AVBT might preserve more motion than traditional PSF, although the extent of postoperative motion preserved varies with the level of instrumentation. The most marked ROM preservation comes when comparing fusions to L4 and AVBT to L4. Buyuk et al., however (2021) found less promising results with thoracic AVBT. The group assessed spinal motion in 32 patients 1 year after AVBT via radiographic analysis [36]. Thoracic spine segments showed some coronal and sagittal motion over the instrumented levels without showing autofusion at the 1-year follow-up (*p* < 0.0001). These authors noted coronal plane motion was reduced by 77%. Engsberg et al. (2002) evaluated global changes in triplanar spine ROM in 30 patients with AIS following spinal fusion.[37]. Contrary to the compensatory increases suggested by Marks et al., Engsberg found a general decrease in ROM across both fused and unfused regions, as well as globally. This was observed in all planes (coronal, sagittal, and transverse). Engsberg found global forward ROM to be 37.9° which dropped to 24.5° at 12 months postoperatively. Notably, there was no compensatory increase in ROM in the unfused segments even in lateral bending, suggesting these areas do not necessarily adapt by increasing movement to compensate for the immobilized fused regions. In unfused regions, significant decreases in ROM were noted for left lateral flexion both above and below the fused segments at 24 months post-surgery (*p* < 0.05). Global measurements also showed significant reductions in ROM for lateral flexion and forward flexion (*p* < 0.05). Finally, Yao et al. (2017) characterized segmental curve flexibility in 80 AIS patients who had underwent instrumented PSF. At the 2-year follow-up point, Yao et al. (2017) found that the mean segmental fulcrum bending correction index (FBCI) was 155%, 131%, and 100% in the upper, mid, and lower segments, respectively (*p* < 0.001). Their data suggests that distal curve segments had greater flexibility [38]. A positive correlation was noted between the segmental bending Cobb angle and the segmental FBCI (*p* < 0.05); the varying correlation strengths suggest that flexibility is not homogenous throughout the spine, where different segments exhibit greater flexibility/correctability than others. Yao’s findings regarding segmental flexibility align with those of Marks et al. (2012), who also support the benefit of distributing motion across more spinal segments for preserving ROM.

## Discussion

The primary aim of this study was to evaluate how segmental and global ROM are affected in AIS patients, both with and without surgical intervention. Our review revealed large variability in segmental flexibility and its preservation or loss, with fused spines showing predictable motion loss post-surgery. AVBT preserved slightly more motion compared to PSF, though the long-term clinical significance is unclear. The literature is mixed on whether motion increases in unfused levels post-PSF for AIS, likely due to varied measurement techniques across studies. Consistent, unbiased measurement methods are needed to allow for direct comparisons of findings.

Data consistently showed a periapical loss of flexibility in scoliotic spines, present in both the thoracic and lumbar regions. In non-operative AIS patients, the peri-apical regions were consistently stiff in both areas. This increased rigidity could be attributed to the structural deformity inherent in scoliosis, limiting the mobility of these segments [16, 17]. Interestingly, sites distant from the apex (> 5 segments) exhibited a greater flexibility and compensatory increase in motion compared to controls [31, 32]. This may be a mechanism to maintain global spinal mobility despite rigidity near the apex. This suggests an autoregulatory phenomenon where flexibility increases with distance from the apex to preserve overall spinal motion. The brain recognizes an imbalance in the coronal plane and adjusts spinal alignment to maintain a neutral position, defined by a C7 plumb line, despite scoliosis [39, 40]. Previous literature supports that non-scoliotic segments may adapt to provide greater flexibility to counterbalance the stiff, scoliotic regions [25]. This highlights the body’s natural tendency to maintain sagittal and coronal balance. This concept is crucial for tempering correction of the main thoracic curve to accommodate the proximal thoracic curve and understanding flexibility in scoliosis even before correction [31]. It is unclear if this compensatory flexibility is maintained over time or if the spine stiffens in untreated scoliosis in adulthood. This adaptive response may help mitigate functional limitations imposed by rigid scoliotic segments, allowing patients to perform daily activities more effectively and maintain an optimal upright posture against gravity. This adaptive response may however be limited in AIS patients, as the potential for degenerative disc disease (DDD) and back pain caudal to the fusion is often seen with instrumented fusions. DDD tends to be associated with distal lumbar LIVs, and recently, Lonner et al. (2018) have demonstrated that more than 50% of DD occurred at the second (35.5%) and third (20%) disc caudal to the LIV which may further contribute to back pain, stiffness, and possibly sciatica symptoms if nerve compression occurs due to disc herniation, in some patients [41]. This provides further evidence supporting clinical reccomendations to preserve as many caudal motion segments, with the authors ultimately suggesting to avoid fusion to L4, and maintain the LIV tilt angle below 5° and LIV translation less than 2 cm [41].

We found mixed evidence regarding motion after PSF. As we hypothesized, increasing the number of preserved spinal segments was seen to be strongly associated with greater global ROM which is known to be important for the prevention of later degenerative changes [41]. However, disagreement was noted as far as segmental motion with some studies suggesting that a more distal LIV may result in greater compensatory motion in the segments below the fusion for lateral bending, whereas other studies found loss of motion in all planes distal to the fusion [18, 19, 25, 35, 37]. Authors who obtained the latter findings showed that fusion tended to show a 7-degree global motion loss of flexion motion with every additional LIV fused in PSF, with the greatest motion loss seen with fusions to L4 [35]. A similar study comparing fused and nonfused AIS patients similarly shows that fused patients had a 25% decrease overall in global ROM postoperatively [42]. This finding is interesting and contrary to the non-operative state where segments distal to scoliosis show compensatory gains in motion. When comparing global ROM between patients without scoliosis, untreated AIS, and AIS with PSF, the PSF group displayed significantly reduced global ROM during thoracic and total left axial twists (*p* ≤ 0.048) compared to the non-operative groups [21]. Although reduced ROM after fusion is expected, further investigation into the impact of increasingly distal fusion is needed to understand compensatory distal motion after PSF. Technique variations in measurement between articles may explain some differences, but the opposing findings of Marks and Yao versus Pahys and Engsberg are concerning.

Our limited review of AVBT shows that it offers some preservation of ROM compared to PSF, specifically in the lumbar spine, and the difference was seen most dramatically as LIV approached L4 [35, 36]. Pahys et al. (2022) seem to indicate that AVBT spares roughy 10–15 degrees of ROM over PSF for L1-3 relative to the preop state, and the technique saves around 20–25 degrees at L4 compared to PSF [35]. This is of course under the assumption that AVBT does not fail or need to be revised, which there is still an estimated 25% chance, or cause autofusion when assessed beyond 5 years of follow-up [43]. The clinical significance and long-term outcome of retained motion are currently unknown. While AVBT offers the advantage of preserving motion compared to PSF, the tradeoffs between more reliable correction and lower reoperation rates with PSF versus greater flexibility and faster recovery with sAVBT require further investigation. Furthermore, the clinical significance of global ROM loss in selective thoracic PSF is debatable, as it may be negligible given long-term benefits offered to patients. Ten year prospective data on PSF show excellent patient outcomes, a low revision rate and excellent patient satisfaction [44]. Newton et al. (2020) further showed that AIS patients report quality of life (QOL)10 years after surgery to be substantially better than patients who refused surgery, with a low 10-year chance of revision (7.5%) [45]. At 2-year follow-up, only 76% of thoracic idiopathic scoliosis AVBT patients had a residual curve of < 35° compared with 97.4% of PSF patients [46]. The literature further shows that PSF, when compared to AVBT, offers greater corrective maintenance at a 2-year period and beyond, while AVBT shows complications such as broken tethers, revision surgery and even damaged viscera [46–48]. A meta-analysis of AVBT versus PSF showed that pooled complication rates for VBT were 26% compared to 2% in PSF, and a revision rate of 24.7% for AVBT compared to 1.8% in PSF [43]. Vokes et al. (2021) and Lander et al. (2022) further studied a mimimum 40-year follow-up to Harrington rod instrumented PSF, finding normal self-reported health-related QOL compared with the age-matched population [9, 49]. AVBT, though promising, has not supplanted PSF as the gold standard in AIS surgery due to the mentioned issues and unclear clinical benefits over PSF in selective thoracic fusion cases. AVBT is a potential solution for children with growth remaining, especially when fusion to L4 is the alternative. Indications for AVBT in the thoracic spine are rare, and while lumbar tethering to L4 shows promise, further research is needed to determine its optimal use. A hybrid procedure, with lumbar tethering and thoracic spine fusion, may be the solution for treating Lenke 3, 4, and 6 curve patterns requiring long fusions to L4.

This systematic review has several limitations. The qualitative approach was necessary due to heterogenous data and low-quality individual studies, limiting the conclusions. Segment-specific ROM reporting was fragmented, hindering comparisons between surgery types and operative vs. nonoperative patients. Measurement methods for ROM varied greatly among studies. Some studies relied on radiographic methods, which provide precise internal measurements but may not fully capture dynamic spinal motion. In contrast, other studies employed surface tracking or physical exams, which allow for real-time, functional assessments of movement but may lack the precision of radiographic imaging. These differences may contribute to variability in the reported findings and should be considered when interpreting the current literature. The differences between some studies showing hypermobility below fused segments versus others showing diminished mobility may stem from variations in measurement methods and postoperative management. Factors like return-to-activity timing and the level of permitted physical activity likely influence these outcomes. Future prospective studies could control for these variables to provide clearer insights into their impact on mobility. All included articles were observational, many of low quality, reducing data certainty. Despite these limitations, the review builds on previous literature on global ROM, provides preliminary surgical guidance, and calls for further research. More controlled, homogeneous, and high-quality studies are needed to guide surgeon decision-making and clarify the benefits of motion-sparing technology.

## Conclusion

This systematic review identifies the current knowledge on motion in fused and unfused adolescent idiopathic scoliotic spines and the effects of PSF and AVBT. Findings highlight the balance between preserving ROM and achieving structural correction in AIS treatment. The scoliotic spine naturally exhibits periapical stiffness, with compensatory increased ROM in non-operative distal segments. Fused spines do not adapt to increase movement distant from the fusion, except in lateral bending, with mixed results in the literature. Postoperative global motion reduction reflects fusion constraints, especially in the lumbar spine, where segmental motion decreases with more distal LIV. Preserving more spinal segments is associated with greater global ROM, important for preventing degenerative changes. AVBT tends to preserve more motion post-surgery compared to PSF, particularly in the lowest lumbar segments, notably L4. This review highlights limited segmental motion in the thoracic spine and the lack of data on motion preservation with motion-sparing surgery for AIS. Additional studies, like Pahys et al., are needed to confirm lumbar motion preservation with AVBT, crucial for pediatric spine surgeons counseling families. AVBT remains a candidate solution for the lumbar spine in pediatric patients requiring fusion to L4, but long-term follow-up and improvements in technique and materials are necessary, emphasizing shared decision-making.
